# Supplementary material for: Systematic review on cost-effectiveness analysis of school-based oral health promotion program
Source: PLoS One. 2023 Apr 20;18(4):e0284518. doi: 10.1371/journal.pone.0284518 (PMC10118196; doi:10.1371/journal.pone.0284518)
Supplement: S1 Table — (PDF) [file pone.0284518.s001.pdf]

S1 Table. Excluded studies list

No	Title	Exclusion Criteria	Reason for exclusion	Authors
1	Cost-effectiveness study of a school-based sealant program	Not population of interest	Population age was not mentioned	Werner, CW; Pereira, AC; Eklund, SA
2	Costs and cost-effectiveness of different caries-preventive measures in youth dental care in Sweden	Not population of interest	The population of the study was adolescent	Oscarson, Nils;
3	Long-Term Cost-Effectiveness through the Dental-Health FRAMM Guideline for Caries Prevention	Not population of interest	The population of the study were 12-15 y.o children (Junior High School age)	Davidson, Thomas; Bergström, Eva-Karin; Husberg, Magnus; Moberg Sköld, Ulla;
4	Cost-effectiveness of a telephone-delivered education programme to prevent early childhood caries in a disadvantaged area: a cohort study	Not population of interest	The population of the study were <5 y.o children (pre-school)	Pukallus, M; Plonka, K; Kularatna, S; Gordon, L; Barnett, AG; Walsh, L; Seow, WK
5	Cost-effectiveness of a disease management program for early childhood caries	Not population of interest	The population of the study were 2-5 y.o children (pre-school)	Samnaliev, M; Wijeratne, R; Kwon, EG; Ohiomoba, H; Ng, MW
6	Assessing the cost-effectiveness of a fluoride varnish programme in Chile: The use of a decision analytic model in dentistry	Not population of interest	The population of the study were <5 y.o children (pre-school)	Palacio, R; Shen, J; Vale, L; Vernazza, CR
7	Cost-effectiveness analysis of the atraumatic restorative treatment-based approach to managing early childhood caries	Not population of interest	The population of the study were <5 y.o children (pre-school)	Tonmukayakul, U; Arrow, P
8	Cost-Effectiveness of Caries Prevention in Practice: A Randomized Controlled Trial	Not population of interest	The population of the study were <5 y.o children (pre-school)	O'Neill, C; Worthington, HV; Donaldson, M; Birch, S; Noble, S; Killough, S; Murphy, L; Greer, M; Brodison, J; Verghis, R; Tickle, M
9	Relative cost-effectiveness of home visits and telephone contacts in preventing early childhood caries	Not population of interest	The population of the study were <5 y.o children (pre-school)	Koh, R; Pukallus, M; Kularatna, S; Gordon, LG; Barnett, AG; Walsh, LJ; Seow, WK
10	A randomised controlled trial to measure the effects and costs of a dental caries prevention regime for young children attending primary care dental services: the Northern Ireland Caries Prevention In Practice (NIC-PIP) trial	Not population of interest	The population of the study were <5 y.o children (pre-school)	Tickle M, O'Neill C, Donaldson M, Birch S, Noble S, Killough S, Murphy L, Greer M, Brodison J, Verghis R, Worthington HV.

11	An evaluation of a primary preventive dental programme in non-fluoridated areas of Victoria, Australia	Not population of interest	The population of the study were 12-15 y.o children (Junior High School age)	Morgan MV, Campain AC, Crowley SJ, Wright FA.
12	Caries and costs: an evaluation of a school-based fluoride varnish programme for adolescents in a Swedish region	Not population of interest	The population of the study were 12-15 y.o children (Junior High School age)	Bergström EK, Lingström P, Hakeberg M, Gahnberg L, Sköld UM.
13	Comparing the costs of three sealant delivery strategies	Not population of interest	The population of the study were 12-15 y.o children (Junior High School age)	Griffin SO, Griffin PM, Gooch BF, Barker LK.
14	Home Visits and Telephone Contacts for Preventing Early Childhood Caries could be Cost Effective	Not population of interest	The population of the study were <5 y.o children (pre-school)	Griffin SO, Griffin PM.
15	Caries-preventive effect on primary and permanent teeth and cost-effectiveness of an NaF tablet preschool program	Not population of interest	The population of the study were <5 y.o children (pre-school)	Widenheim J., Birkhed D.,
16	Cost-effectiveness Analysis of the Dental RECUR Pragmatic Randomized Controlled Trial: Evaluating a Goal-oriented Talking Intervention to Prevent Reoccurrence of Dental Caries in Children	Not population of interest	The article is not yet published	Victory, E; Rhiannon, ET; Girvan, B; Pauline, A; Cynthia, PM
17	Seal or varnish? Cost-effectiveness of fissure sealants versus fluoride varnish in preventing dental decay in children	Not exposure of interest	Not a school-based program	Humphreys, I; Chestnut, I; Fitzsimmons, D; ,,,20,9,A867,2017,Elsevier
18	Cost-effectiveness and efficacy of fluoride varnish for caries prevention in South African children: A cluster-randomized controlled community trial	Not exposure of interest	Not a school-based program	Effenberger, S; Greenwall, L; Cebula, M; Myburgh, N; Simpson, K; Smit, D; Wicht, MJ; Schwendicke, F
19	Modeling an economic evaluation of a salt fluoridation program in Peru	Not exposure of interest	Not a school-based program	Mariño RJ, Fajardo J, Arana A, Garcia C, Pachas F.
20	School-Based Dental Sealant Programs Prevent Cavities And Are Cost-Effective	Not exposure of interest	Not a school-based program	Griffin S, Naavaal S, Scherrer C, Griffin PM, Harris K, Chattopadhyay S.
21	A cost-effectiveness analysis of community water fluoridation for schoolchildren	Not exposure of interest	Not a school-based program	Cronin J., Moore S., Harding M., Whelton H., Woods N.,
22	Cost-effectiveness of an experimental caries-control regimen in a 3.4-yr randomized clinical trial among 11-12-yr-old Finnish schoolchildren	Not exposure of interest	Not a school-based program	Hietasalo, P; Seppa, L; Lahti, S; Niinimaa, A; Kallio, J; Aronen, P; Sintonen, H; Hausen, H
23	Cost-effectiveness simulation of a universal publicly funded sealants application program	Not exposure of interest	Not a school-based program	Bertrand, E; Mallis, M; Bui, NM; Reinharz, D

24	Cost-effectiveness of managing cavitated primary molar caries lesions: A randomized trial in Germany	Not exposure of interest	Not included in preventive measures (curative)	Schwendicke F., Krois J., Splieth C.H., Innes N., Robertson M., Schmoeckel J., Santamaria R.M.,
25	Cost effectiveness and dental caries prevention	Not exposure of interest	Not a school-based program	van Loveren, C;
26	The cost-effectiveness of a two-step blood pressure screening programme in a dental health-care setting	Not exposure of interest	Not included in preventive measures of interest (blood test)	Andersson, Helen; Svensson, Mikael; Bergh, Håkan;
27	The cost-effectiveness of three interventions for providing preventive services to low-income children	Not exposure of interest	Not a school-based program	Johnson, B; Serban, N; Griffin, PM; Tomar, SL
28	Methods and preliminary findings of a cost-effectiveness study of glass-ionomer-based and composite resin sealant materials after 2yr	Not exposure of interest	Not a school-based program	Goldman, AS; Chen, X; Fan, MW; Frencken, JE
29	Cost-effectiveness of extending the coverage of water supply fluoridation for the prevention of dental caries in Australia	Not exposure of interest	Not a school-based program	Cobiac, LJ; Vos, T
30	Cost-effectiveness, in a randomized trial, of glass-ionomer-based and resin sealant materials after 4yr	Not exposure of interest	Not a school-based program	Goldman, AS; Chen, X; Fan, MW; Frencken, JE
31	Cost-Effectiveness of Pit-and-Fissure Sealants on Primary Molars in Medicaid-Enrolled Children	Not exposure of interest	Not a school-based program	Chi, DL; van der Goes, DN; Ney, JP
32	Cost-effectiveness of child caries management: a randomised controlled trial (FiCTION trial)	Not exposure of interest	Not a school-based program	Homer, T; Maguire, A; Douglas, GVA; Innes, NP; Clarkson, JE; Wilson, N; Ryan, V; McColl, E; Robertson, M; Vale, L
33	Effectiveness and Cost-Benefit of an Elementary School-Based Telehealth Program	Not exposure of interest	Not an oral health promotion and prevention program	Long, MW; Hobson, S; Douge, J; Wagaman, K; Sadlon, R; Price, OA
34	Applying cost analysis methods to school-based prevention programs	Not exposure of interest	Not an oral health promotion and prevention program	Chatterji P, Caffray CM, Jones AS, Lillie-Blanton M, Werthamer L.
35	Children's incremental dental care program: an overview of the southeast Tennessee-northwest Georgia project	Not exposure of interest	Not a school-based program	Lewis GP, Monroe GF.
36	Costs and Resource Use Among Child Patients Receiving Silver Nitrate/Fluoride Varnish Caries Arrest	Not exposure of interest	Not a school-based program	Hansen RN, Shirtcliff RM, Dysert J, Milgrom PM.
37	Value for money: economic evaluation of two different caries prevention programmes compared with standard care in a randomized controlled trial	Not exposure of interest	Not a school-based program	Vermaire JH, van Loveren C, Brouwer WB, Krol M.

38	An Evaluation of the Cost-effectiveness of School Dental Treatment Programs in Contrast to Private Dental Practices	Not exposure of interest	Not included in preventive measures (curative)	Greco, Eugene A;
39	Cost—effectiveness of topically applied fluoride: In"" Relative efficiency of methods of caries prevention in dental public health	Not exposure of interest	Not a school-based program	Heifetz, SB;
40	Cost-effectiveness of Preventive Oral Health Care in Medical Offices for Young Medicaid Enrollees	Not exposure of interest	Not a school-based program	Stearns, SC; Rozier, RG; Kranz, AM; Pahel, BT; Quinonez, RB
41	Cost analysis of 3 years of topical fluoride application	Not exposure of interest	Not a school-based program	Vrbic V, Kosmelj B.
42	Cost-Effectiveness Analysis of Dental Sealants versus Fluoride Varnish in a School-Based Setting	Not outcome of interest	Did not mention the incremental cost and incremental effect	Neidell, M; Shearer, B; Lamster, IB
43	The cost and effectiveness of school-based preventive dental care	Not outcome of interest	Did not mention the incremental cost and incremental effect	Klein, Stephen P; Bohannan, Harry M; Bell, Robert M; Disney, Judith A; Foch, Craig B; Graves, Richard C; ,.,,75,4,382-391,1985,American Public Health Association
44	A study of pit and fissure sealing in the School Dental Service	Not outcome of interest	Did not mention the incremental cost and incremental effect	Hunter PB.
45	An economic evaluation of a publicly funded dental prevention programme in regional and rural Victoria: an extrapolated analysis	Not outcome of interest	Did not mention the incremental cost and incremental effect	Crowley SJ, Campain AC, Morgan MV.
46	Costs of a school-based dental mobile service in South Africa	Not outcome of interest	Did not mention the incremental cost and incremental effect	Molete MP, Chola L, Hofman KJ.
47	Costs of caries therapy and prevention in a school	Not outcome of interest	Did not mention the incremental cost and incremental effect	Marci F, Antenucci F, Giannoni M.
48	Implementing a Patient-Centered and Cost-Effective School-Based Oral Health Program	Not outcome of interest	Did not mention the incremental cost and incremental effect	Mason M, Gargano L, Kumar A, Northridge ME.
49	Modelling lifelong costs of caries with and without fluoride use	Not outcome of interest	Did not mention the incremental cost and incremental effect	Splieth CH, Flessa S.
50	Analysis of the costs of school-based mouthrinsing programs	Not outcome of interest	Did not mention the incremental cost and incremental effect	Doherty NJ, Martie CW.

51	Cost-analysis of school-based fluoride varnish and fluoride rinsing programs	Not outcome of interest	Did not mention the incremental cost and incremental effect	Sköld UM, Petersson LG, Birkhed D, Norlund A.
52	Costs of school-based mouthrinsing in 14 demonstration programs in USA	Not outcome of interest	Did not mention the incremental cost and incremental effect	Doherty NJ, Brunelle JA, Miller AJ, Li SH.
53	Economic specification of cost estimates in dental programs	Not outcome of interest	Did not mention the incremental cost and incremental effect	Doherty NJ, Crakes GM.
54	Effect of fluoridation on the cost of dental treatment among urban Scottish schoolchildren	Not outcome of interest	Did not mention the incremental cost and incremental effect	Downer MC, Blinkhorn AS, Attwood D.
55	How are economic evaluations carried out for primary caries prevention strategies?	Not outcome of interest	Did not mention the incremental cost and incremental effect	Quach H.
56	Prediction of costs in a selective caries prevention programme	Not outcome of interest	Did not mention the incremental cost and incremental effect	Helfernstein U, Steiner M.
57	Study protocol of the cost-effectiveness comparison of two preventive methods in the incidence of caries: A randomized, controlled clinical trial	Not original research	Research protocol	Fernández-Barrera M.Á., Lara-Carrillo E., Scougall-Vilchis R.J., Pontigo-Loyola A.P., Ávila-Burgos L., Casanova-Rosado J.F., Casanova-Rosado A.J., Minaya-Sánchez M., Medina-Solís C.E.,
58	Cost-effectiveness of dental hygiene care	Not original research	Web page	Notgarnie, Howard M;
59	The Cost-effectiveness of Dental Preventive interventions (fissure sealant & flouride varnish) in Elementary School Children	Not original research	Systematic review	Jafarzadeh, Dariush;
60	Dental Home Visits for Caries Prevention Among Preschool Children: Protocol for a Cost-Effectiveness Analysis on a Randomized Control Trial	Not original research	Research protocol	Andiesta, NS; Hamid, MA; Lee, KKC; Pau, A
61	Effectiveness, cost-effectiveness and cost-benefit of a single annual professional intervention for the prevention of childhood dental caries in a remote rural Indigenous community	Not original research	Research protocol	Lalloo, R; Kroon, J; Tut, O; Kularatna, S; Jamieson, LM; Wallace, V; Boase, R; Fernando, S; Cadet-James, Y; Scuffham, PA; Johnson, NW
62	Protocol for Seal or Varnish? (SoV) trial: a randomised controlled trial to measure the relative cost and effectiveness of pit and fissure sealants and fluoride varnish in preventing dental decay	Not original research	Research protocol	Chestnutt, IG; Chadwick, BL; Hutchings, S; Playle, R; Pickles, T; Lisles, C; Kirkby, N; Morgan, MZ; Hunter, L; Hodell, C; Withers, B; Murphy, S; Morgan-Trimmer,

				S; Fitzsimmons, D; Phillips, C; Nuttall, J; Hood, K
63	Analysis of the cost effectiveness of 2 school preventive programs [Análisis de costo-eficacia de dos programas preventivos escolares.]	Full text not available online		Dono R., de Canton L.P., Argentieri A.,
64	Cost effectiveness analysis of 3 alternative community programs in the preventive use of fluoride for dental caries prevention in Cataluña [Análisis coste-efectividad de tres programas comunitarios alternativos de uso preventivo del flúor para la prevención de la caries dental en Cataluña.]	Full text not available online		Salleras L., Bohigas L., Cuenca E., Martinez-Carretero J.M., Manau C.,
65	Fluoride in the prevention of dental caries. A tentative cost-benefit analysis. 5. The cost-effectiveness of professionally-administered topical applications of fluoride solutions	Full text not available online		Davies, GN;
66	Cost-effectiveness analysis of a school-based dental sealant program for low-socioeconomic-status children: A practice-based report	Full text inaccessible		Zabos G.P., Glied S.A., Tobin J.N., Amato E., Turgeon L., Mootabar R.N., Nolon A.K.,
67	A comparative study on cost-effectiveness between resin and glass-ionomer used as pit and fissure sealant in mobile dental service for school children at Municipality school, Buriram province, Thailand	Full text inaccessible		Thipsoonthornchai, Jeerasak;
68	COST-EFFECTIVENESS OF A PREVENTIVE PROGRAM FOR NATIONAL SCHOOLCHILDREN WITHIN THE HEALTH BOARD DENTAL SERVICES	Full text inaccessible		BELL, CJ; BUCKLEY, K; OMULLANE, DM; ONEILL, BN;
69	Examination of Oral Health Prevention Strategy of Dental Sealants in Children: Cost-effectiveness Analysis and Policy Implications	Full text inaccessible		Hodges, Virginia Anne;
70	The cost-effectiveness of the prevention of dental caries among primary school students	Full text inaccessible		Meerat, B; Lounkeaw, K;
71	The cost-effectiveness evaluation of 2 caries prevention strategies compared with the standard approach [Een economische evaluatie van 2 cariëspreventieve strategieën vergeleken met standaardzorg]	Article is not in English	Dutch	Vermaire J.H., van Loveren C., Brouwer W.B., Krol M.,
72	Cost-effectiveness analysis of preventive methods for occlusal surface according to caries risk: results of a controlled clinical trial	Article is not in English	Portuguese	Tagliaferro, EPD; Marinho, DS; Pereira, CCD; Pardi, V; Ambrosano, GMB; Meneghim, MD; Pereira, AC
