# Supplementary material for: Systematic review on cost-effectiveness analysis of school-based oral health promotion program
Source: PLoS One. 2023 Apr 20;18(4):e0284518. doi: 10.1371/journal.pone.0284518 (PMC10118196; doi:10.1371/journal.pone.0284518)
Supplement: Attachment — Submitted filename: Review PLOSone.docx [file pone.0284518.s004.docx]

**Comments to the authors:**

Thank you for addressing a very important topic for the economic evaluation of different preventive and oral health promotion programs in elementary school-going children. The manuscript is adequately written however, there is some room for improvement.

Attached below are the comments for your reference:

**Major points:**

- The manuscript is quite wordy and should be significantly reduced in length.
- There are several formatting errors, especially the referencing. References should be cited at the end of the statement and after full stop.
- Try to write the manuscript in third-person past tense and get it reviewed by someone proficient in English writing.

**Introduction:**

- Very well written; writing is supported by evidence.
- Line 48-51: There is redundancy, can be rephrased to a single statement.
- Line 64: Reference should never be in the middle of the statement.
- Some details about cost-effectiveness analysis and its utility can be added here in the introduction.

**Methods:**

- Fairly adequate description of the methodology; clear research question and PICOs model.
- The search terms are inadequate. Should have included more terms like incremental cost, incremental effects, ICER, QALYs, DALYs etc.
- Line 116-118: This information is not needed.
- Line 173-174: The authors have used JBI checklist for systematic review of economic evaluations however, they should have also used the GRADE tool to determine the certainty in evidence.

**Results:**

- Line 229-230: Amongst the included studies, only a single study has reported outcome in the form of DALYs which is an outcome measure for cost-utility analysis. The authors could have excluded this study to reduce heterogeneity. Else they could have expanded their search to include QALYs, DALYs, and cost-utility analysis along with some other relevant terms to yield more comprehensive results.
- Table 3 provides all the information regarding cost of interventions as well as control, however, there is no clear information in text as to what exactly the control group was because there is some cost that is mentioned for control group as well in table 3.
- The authors did not report whether the included studies were reported according to health economics guidelines e.g., CHEERS checklist.

**Discussion:**

- Too lengthy discussion
- Findings should be summarized, too much extra information.
- Line 302-314: Too wordy, can be made brief.
- Line 322-330: Too much information about a cost-effectiveness analysis, can be removed from here and included in the introduction.
- Line 427-432: Not required.

**Limitations:**

- The authors could have mentioned another limitation as the majority of included studies were model-based hence the results could not be adequately generalizable; this is an inherent limitation of cost-effectiveness analysis based on analytic modeling approach as the data on which the model is built may be biased in some way or the other thus, limiting the generalizability of the findings.
